# Differential effects of short- and long-term treatment with mepolizumab on eosinophil kinetics in blood and sputum in eosinophilic asthma

**DOI:** 10.1016/j.isci.2021.102913

**Published:** 2021-07-28

**Authors:** Marwan Hassani, Tamar Tak, Corneli van Aalst, Saar van Nederveen, Kiki Tesselaar, Nienke Vrisekoop, Leo Koenderman

**Affiliations:** 1 Center for Translational Immunology, University Medical Center Utrecht, Heidelberglaan 100, 3584CX Utrecht, The Netherlands; 2 Department of Respiratory Medicine, University Medical Center Utrecht, Heidelberglaan 100, 3584CX Utrecht, The Netherlands; 3 Department of Respiratory Medicine, HAGA Hospital, 2545AA The Hague, The Netherlands

**Keywords:** Health sciences, Immunology, Respiratory medicine, Clinical medicine, Drugs

## Abstract

Mepolizumab (anti-IL-5) is a successful biological for treatment of T2/eosinophilic asthma by blocking the IL-5-eosinophil axis. The kinetics of human eosinophils in blood and sputum was determined to better understand the underlying mechanism(s). Pulse-chase labeling was performed with 6,6-^2^H_2_-glucose in patients with asthma after short term (4 days) and long term (84 days) treatment with mepolizumab (n = 10) or placebo (n = 10). The retention time of eosinophils in sputum was longer than in blood. Treatment with mepolizumab induced a fast and long-lasting eosinopenia with no reduction of eosinophil progenitors. The retention time of eosinophils in blood was delayed only after short-term treatment. This leads to the hypothesis that IL-5 increases the number of *IL-5-responsive* progenitors and potentiates homing to the tissues, leading to reactive eosinophilia. Long-term treatment is associated with low numbers of IL-5-independent eosinophils in blood and tissues. Therefore, long-term treatment with mepolizumab restores the kinetics of eosinophils as normally found in homeostasis.

## Highlights

• Anti-IL-5 (mepolizumab) treatment leads to inhibition of reactive eosinophilia
• Reactive blood eosinophils have a high retention time in the absence of IL-5
• Eosinophils are long lived in the sputum of eosinophil asthmatics
• Anti-IL-5 reduces proliferating progenitors rather than inhibiting differentiation

## Graphical abstract

**Figure:**
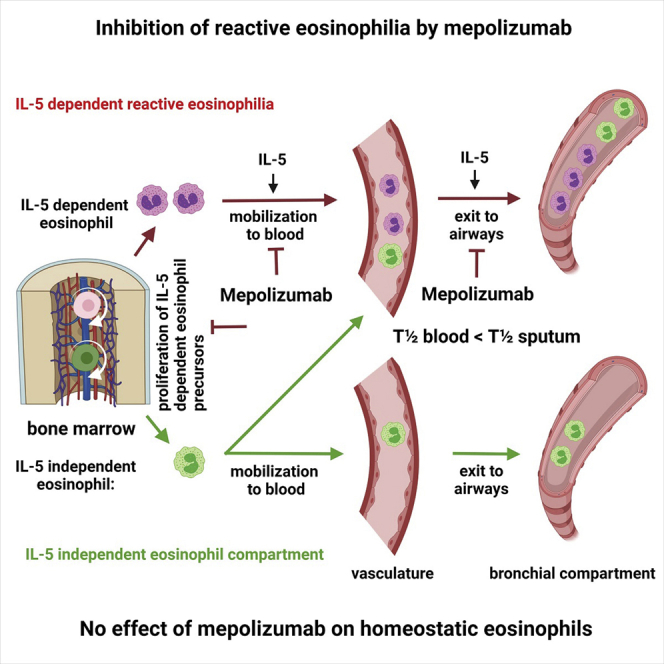

## Introduction

Asthma is a chronic respiratory disease characterized by bronchial inflammation leading to reversible airways obstruction and airway hyperresponsiveness. The disease is characterized by episodes of progressive shortness of breath, cough, wheezing, and chest tightness (Bousquet et al., 2010). It is a very common disorder affecting around 300 million individuals of all ages worldwide (To et al., 2012). This makes asthma a major cause of morbidity that leads to a high utilization of health resources (Braman, 2006). Nowadays, asthma is no longer considered as a single entity but rather as a disease with multiple endotypes (Haldar et al., 2008). It can, for example, be classified as atopic or nonatopic, based on the presence or absence of specific IgE to inhaled allergens, respectively (Vijverberg et al., 2013). Another way to classify asthma is based on the absence or presence of eosinophilic inflammation. Eosinophilic asthma (EA) is characterized by the presence of a significant number of sputum and/or blood eosinophils, whereas noneosinophilic asthma is characterized by either the presence of high numbers of sputum neutrophils (neutrophilic) or very few inflammatory sputum cells (paucigranulocytic) (Simpson et al., 2006). The need for asthma classification has gained importance with the development of novel and specific immune-based therapies that are only efficacious in particular subgroups of asthma (Haldar et al., 2008). This is particularly true for EA, as IL-5-targeted therapy proved to be only efficacious in asthma characterized by eosinophilic inflammation (Haldar et al., 2009). Mepolizumab and reslizumab are both humanized monoclonal antibodies (Mabs) that bind and neutralize circulating IL-5 (Hassani and Koenderman, 2018). Benralizumab on the other hand is a Mab that binds directly to the IL-5 receptor (IL-5Rα/CD125) which leads to the induction of cell death by antibody-dependent cellular cytotoxicity (ADCC) (Kolbeck et al., 2010). In humans, the responsiveness to IL-5 in terms of survival and activation is restricted to basophils and eosinophils (Takatsu, 2011). Although there are preliminary studies that suggest the presence of IL-5Rα on ILC2s (Smith et al., 2016) and airway epithelial cells (Barretto et al., 2020). The precise functions of IL-5 in humans have not been completely identified because most studies have either been performed *in vitro* or in animal models *in vivo* (Hassani and Koenderman, 2018). Murine studies suggest that IL-5 is important in the induction of reactive eosinophilia during allergen provocations and parasitic infections (Foster et al., 1996). The role of IL-5 in homeostatic eosinopoiesis is much less clear as IL-5-knockout (KO) mice and mice with deleted β-common and β-IL-3 genes (no receptors for IL-3, IL-5, and GM-CSF) have terminally differentiated eosinophils in the peripheral blood (Sanderson, 1992) (Broughton et al., 2015).

Treatment with mepolizumab leads to a fast reduction in the number of eosinophils and their progenitors (myelocytes and metamyelocytes) in blood and bone marrow, but there is no complete depletion (Menzies-Gow et al., 2003). On the other hand, resident eosinophils in duodenal tissue are not affected by such treatment (Conus et al., 2010) and high doses of mepolizumab reduced only 50% of the eosinophil numbers in bronchial biopsies even after 10 weeks of treatment (Flood-Page et al., 2003). Furthermore, the number of early progenitors (CD34/IL-5Rα) in the bone marrow was not affected by treatment with mepolizumab (Menzies-Gow et al., 2003). These data suggest that targeting IL-5 attenuates the number of early proliferating eosinophil progenitors in humans rather than an effect on their differentiation *per se*, as mature eosinophils are present both in tissue (bone marrow and duodenum) and blood. A good explanation for the relatively low effect of mepolizumab in tissues has not been provided yet. For example, it is unknown whether the residual eosinophils after anti-IL-5 treatment are longer lived in the tissue or that the low amount of eosinophils produced (independently of IL-5) preferentially home to the bronchial airways. Alternatively, it is possible that tissue eosinophils are not IL-5 dependent owing to low or no expression of IL-5Rα (Liu et al., 2002).

Studies on the life cycle of eosinophils are limited. The life cycle refers to four phases in the life of an eosinophil: 1. proliferation and differentiation in the bone marrow, 2. time (half-life) in the peripheral blood, 3. the time that the cells stay in and exit from the tissues, and 4. the moment of programmed cell death. The consensus in literature is that eosinophils and basophils are very short lived (half-life of 2–3 days and 3–4 days in peripheral blood, respectively) (Steinbach et al., 1979)^,^ (Cartwright et al., 1964). They are continuously produced by the bone marrow and die by apoptosis in the tissue (Geering et al., 2013). These insights in humans are based on *ex vivo* labeling of cells with (radioactive) tracers before reinfusion into the body or by toxic tracers *in vivo* (Dale et al., 1976; Lukawska et al., 2014; Parwaresch et al., 1976). However, this methodology significantly affects the homing behavior of cells as *in vitro* manipulation activates leukocytes and thus leads to underestimation of the half-lives of nontouched cells (Farahi et al., 2012).

In this study we used deuterated glucose to label all dividing cells *in situ* (Busch et al., 2007; Macallan et al., 2009) of 20 patients with moderate to severe asthma in order to study the life cycle of eosinophils and basophils in blood and sputum (eosinophils only). Additionally, these patients were treated with either placebo (n = 10) or mepolizumab (n = 10). This is the first report that has reliably determined the effect of treatment with mepolizumab on eosinophil kinetics in humans, as deuterium-labeled glucose (^2^H) labeling allowed labeling of untouched cells *in vivo* without manipulation or utilization of toxic labels (Macallan et al., 1998).

## Results

### Changes in eosinophil and basophil numbers in blood and sputum during treatment with mepolizumab

The mepolizumab and placebo groups (n = 10 for both groups) were comparable for all tested baseline parameters (Table 1). The absolute count of blood eosinophils showed a rapid and steady decline in the mepolizumab group and was significantly lower than that in the placebo-treated patients within 2 days of treatment (Figure 2A). In marked contrast, basophil numbers in the circulation did not show a significant difference in numbers between the mepolizumab and placebo groups (Figure 2B) despite the fact these cells express functional IL-5 receptors (Hassani and Koenderman, 2018). In the sputum, the percentage of eosinophils determined after 42 days was also significantly lower in patients treated with mepolizumab than in patients receiving placebo (Figure 2C). Interestingly, the number of eosinophil progenitors (EoPs) did not show any significant alterations between the two groups in both blood and sputum (Figure S3). Basophil numbers in sputum were very low and could therefore not be reliably enumerated (data not shown).

**Table 1:** Patient characteristics in the placebo and mepolizumab group, including the characteristics of an earlier eosinophilic asthma study (EHLA)

Patient characteristics	Placebo	Mepolizumab	EHLA	p value^a^
N	10	10	9	–
Female (%)	5 (50%)	6 (60%)	4 (44%)	0.89
Age at inclusion, years (IQR)	51 (33–65)	57 (46–62)	56 (40–72)	0.85
BMI, kg/m^2^ (IQR)	26.2 (23.5–34.0)	28.9 (24.6–35.1)	27.2 (23.2–32.2)	0.90
FEV1, L (IQR)	2.51 (1.92–3.79)	2.33 (1.57–3.24)	2.45 (1.47–3.10)	0.72
FEV1,% predicted (IQR)	88.5 (70.8–101.0)	64.0 (60.1–84.3)	73.7 (52.2–96.6)	0.72
ACQ score at inclusion (IQR)	1.9 (1.3–2.3)	2.2 (1.6–2.8)	1.53 (0.14–2.57)	0.87
ACQ score after 80 days (IQR)	1.6 (1.3–3.3)	2.0 (1.1–2.8)	NA	0.87
Blood eosinophil count at inclusion 10^6^/mL, (IQR)	0.49 (0.28–0.80)	0.40 (0.22–0.70)	0.54 (0.20–2.2)	0.78
Blood basophil count at inclusion 10^6^/mL, (IQR)	0.035 (0.027–.070)	0.046 (0.023–0.071)	NA	0.98
Medication score (IQR)^b^	4 (4–4)	4 (3–4)	4 (3–5)	0.99

Data represent median values (interquartile range).

BMI = body mass index; 0 = no medication; 1 = inhaled SABA when required; 2  = low-dose ICS and SABA; 3  = medium-dose ICS  + LABA; 4  = high dose of ICS  + LABA; 5 = high dose of ICS (and LABA) with OCS; FEV1 = forced expiratory volume in 1 second.; SABA = short-acting beta agonists; LABA = long-acting beta agonists; ICS = inhaled corticosteroids; OCS = oral corticosteroids.

a A Kruskall-Wallis test was performed to compare the placebo with the mepolizumab group.

b 5-point ordinal scale based on guidelines of the British Thoracic Society (British Thoracic Society and Scottish Intercollegiate Guidelines Network, 2019).

**Figure 1:**
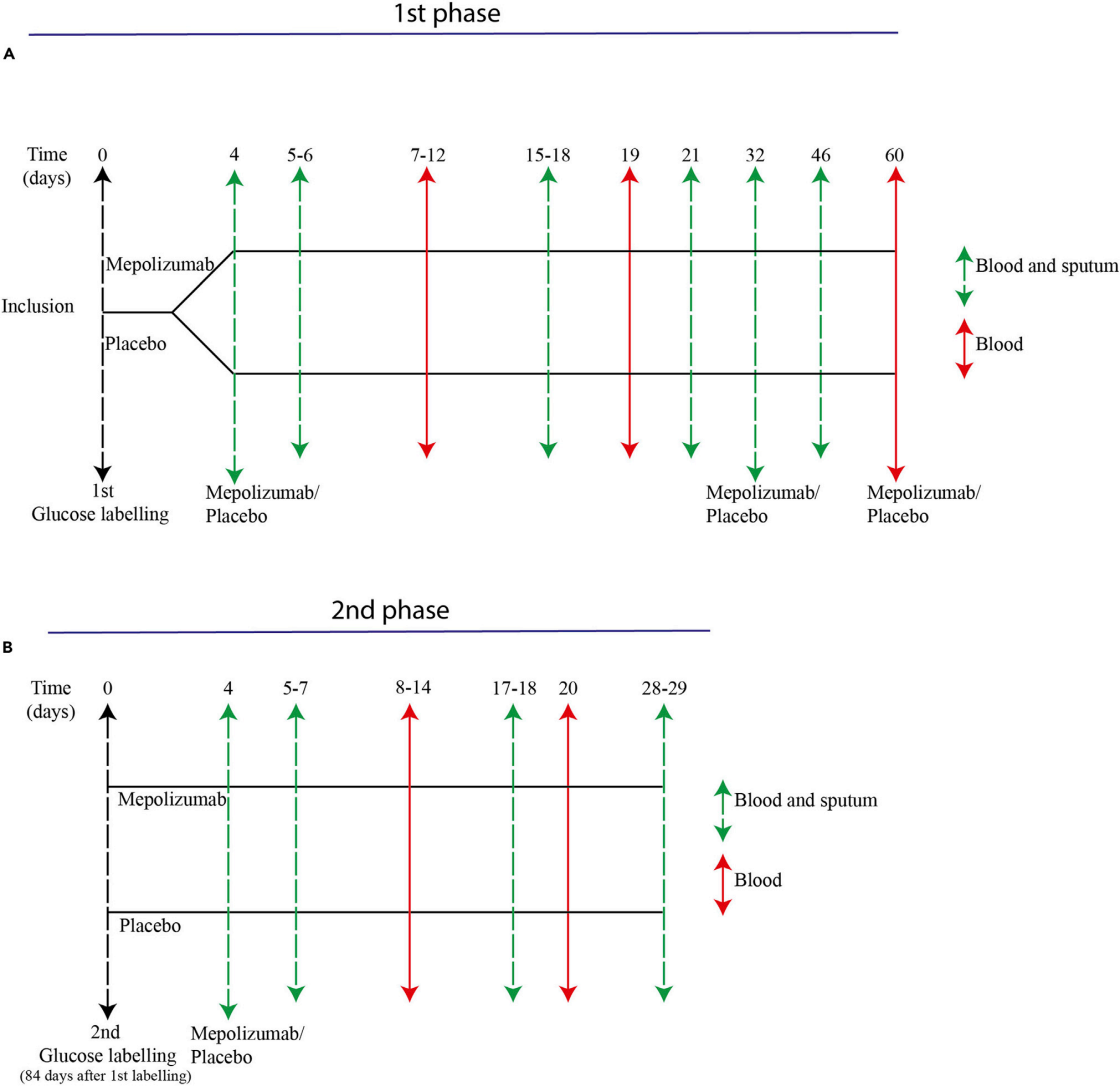
Flowchart of study design (A) During the first phase of the study, 20 patients with asthma were enrolled. At day 0, all patients were labeled with deuterium glucose and baseline blood sample was withdrawn. Thereafter, patients were randomized in a blinded fashion for treatment with mepolizumab (n = 10) or placebo (n = 10). In both groups, only blood (red arrows) or blood and sputum (green arrows) samples were taken on different days, starting from day 4 after label intake. The first phase ended at day 60 after label intake. Mepolizumab or placebo was administered for the first time at day 4 after label intake. This was followed by the second and third administration of mepolizumab or placebo, each time 28 days later (at day 32 or 60, respectively). (B) The second phase started at 84 days after the first label intake. At this day, all patients were again labeled with deuterium glucose and blood was withdrawn. Four days after the second label intake, the fourth and last mepolizumab or placebo dose was administered. Again either blood only (red arrows) or blood and sputum (green arrows) were taken on several following days.

**Figure 2:**
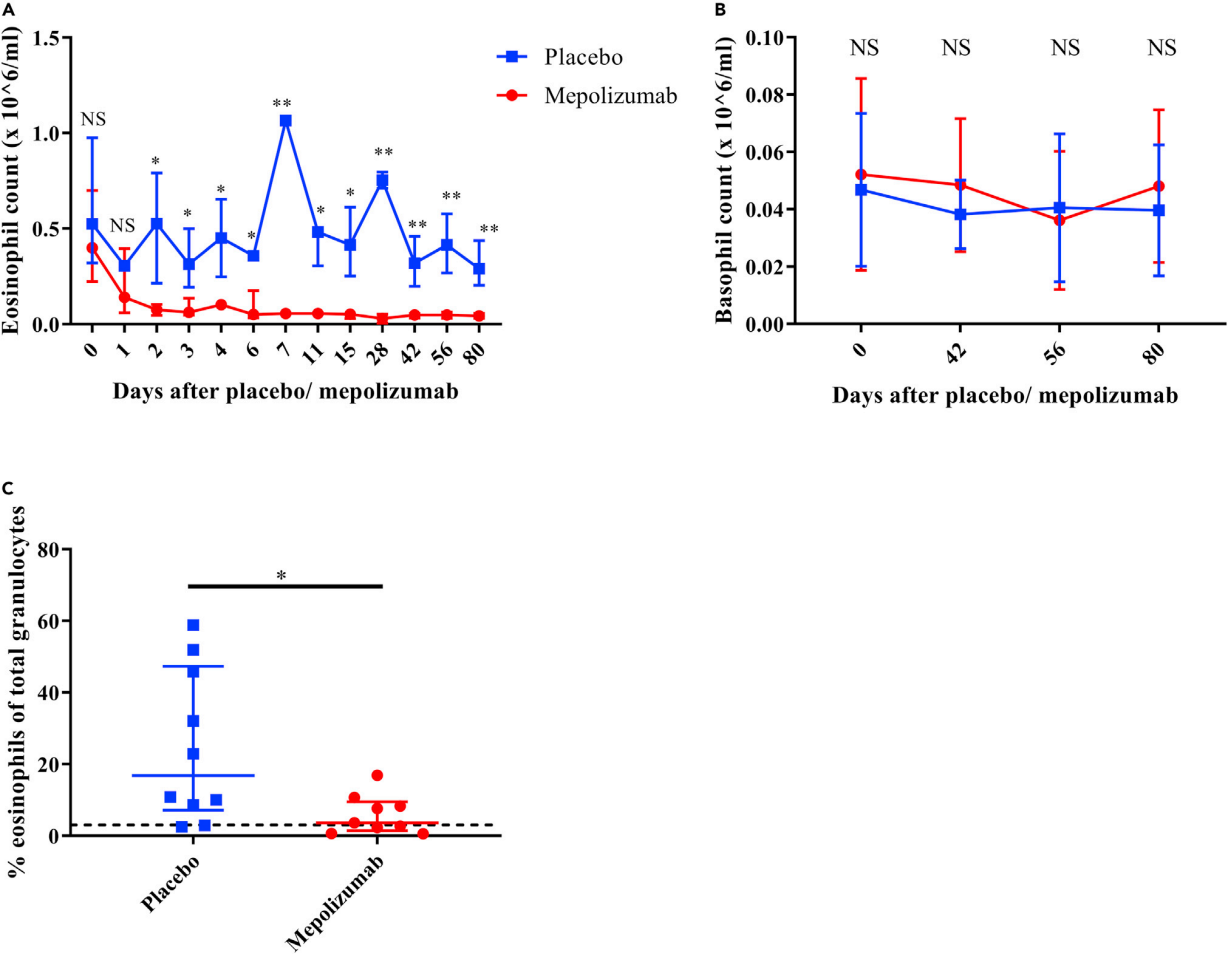
Change of eosinophil and basophil numbers in blood and sputum after treatment with mepolizumab (A) The absolute number of blood eosinophils before and after administration of mepolizumab (red n = 10) or placebo (blue, n = 10). (B**)** The absolute number of blood basophils before and after administration of mepolizumab (red, n = 10) or placebo (blue, n = 10). (C) From 10 patients in the placebo group and 9 patients in the mepolizumab group, the percentage of eosinophils of total granulocytes in the sputum was determined after 42 days after mepolizumab (red) or placebo (blue) treatment. The dotted line is set at 3% (cutoff value for eosinophilic asthma). Median with interquartile range is indicated in each graph. A Mann-Whitney test was performed to compare placebo with mepolizumab at each time point. NS = not significant; ∗p ≤ 0.05 and ∗∗p ≤ 0.01.

### Phenotypic characterization of remaining eosinophils after mepolizumab treatment

**Figure 3:**
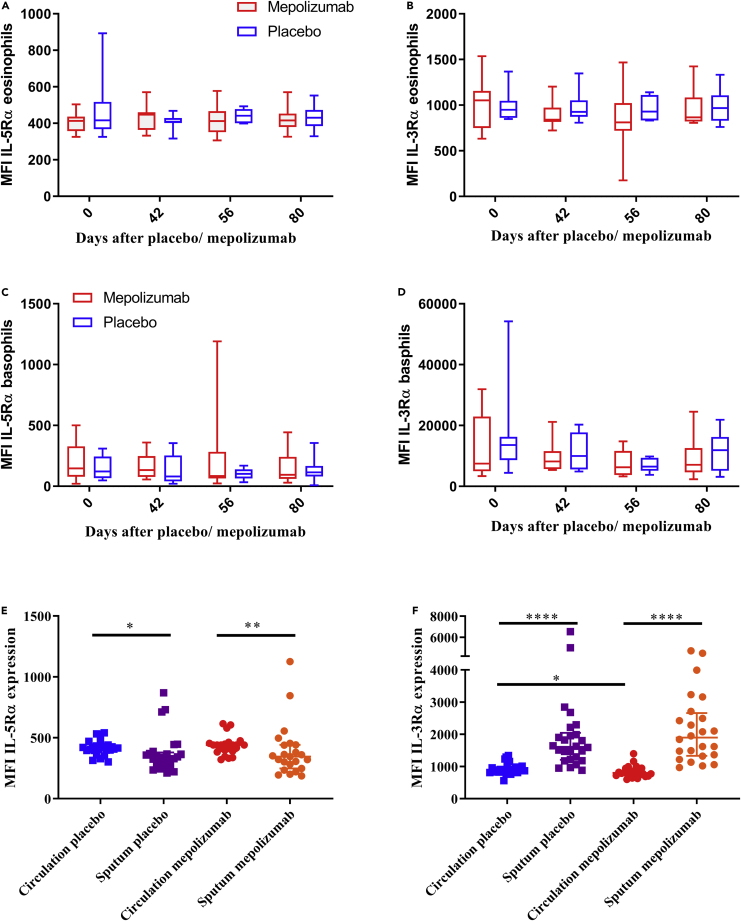
Change of eosinophil and basophil receptor expression in blood and sputum after treatment with mepolizumab Median fluorescence (MFI) intensity on the cell membrane of IL-5Rα (A) and IL-3Rα (B) in blood eosinophils at different time points after either placebo (blue) or mepolizumab (red) administration. Blood basophil expression of IL-5Rα (**C**) and IL-3Rα (D), n = 10 for all groups. The expression of IL-5Rα (E) and of IL-3Rα (F) of the pooled data irrespective of sampling time (>3 days) of treatment with mepolizumab (n = 23) or placebo in blood (n = 25) (red and blue, respectively) and sputum (purple and orange, respectively). Median with interquartile range is indicated in each graph. A Mann-Whitney test was performed to compare placebo with mepolizumab, and a Wilcoxon test was performed to compare blood with sputum. Only statistically significant results are indicated. ∗p ≤ 0.05; ∗∗p ≤ 0.01, and ∗∗∗∗p ≤ 0.0001.

Because the number of eosinophils in blood and sputum clearly decreased after treatment with mepolizumab, we tested the hypothesis that the remaining eosinophils would be phenotypically different. Thus, we compared the expression of several membrane receptors on blood eosinophils and basophils after multiple time points in placebo- and mepolizumab-treated patients. The expression of IL-5Rα and IL-3Rα did not significantly change after treatment on either eosinophils or basophils (Figures 3A–3D). This lack of a difference might be caused by the low number of data per time point. When we pooled the data irrespective of the length of treatment (>3 days), there was a small but significant decrease of IL-3Rα expression on blood eosinophils (3F) after treatment with mepolizumab compared with placebo. This was not found for the expression of IL-5Rα. There was also no difference in IL-5Rα expression in blood and sputum eosinophils after treatment with mepolizumab (Figures 3E and 3F). Regardless of the treatment arm, IL-5Rα expression was lower and IL-3Rα expression was higher on sputum eosinophils than on blood eosinophils.

The expression of CD62L and CD193 was also determined after treatment with mepolizumab or placebo (Figure S4). Interestingly, the expression of L-selectin (CD62L) on eosinophils in blood increased after treatment with mepolizumab (Figure S4A), whereas the expression of CCR-3 (CD193) did not significantly change (Figure S4B).

### The effect of mepolizumab on eosinophil kinetics in blood

**Figure 4:**
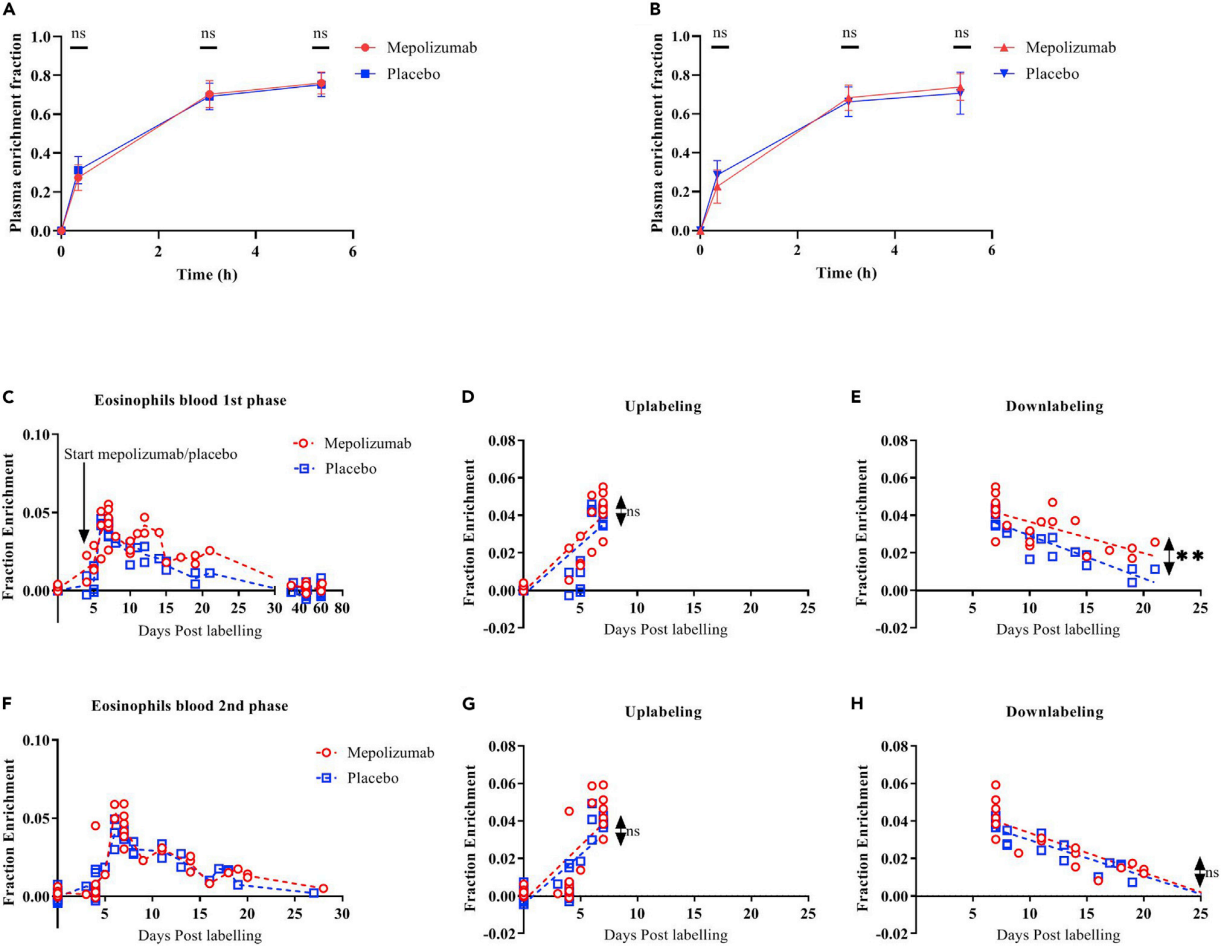
Deuterium enrichment of plasma glucose and DNA of blood eosinophils (A and B) Deuterium enrichment of plasma glucose before and at 3 time points during the intake of oral 6,6-^2^H_2_-glucose to determine the availability of label for each patient in the mepolizumab (red) or placebo (blue) group during the first phase (A) and the second phase (B) of the study. (C**)** The ratio of ^2^H-labeled and total DNA of blood eosinophils was determined before label intake and at several days after label intake for patients in the placebo (blue) and the mepolizumab group (red). (F).This was similarly done for blood eosinophils in both groups after the second label intake. In (A) and (B), median with interquartile range is plotted. In (C) and (F), the individual data points are shown for each time point and a dotted line connects the median values of each day with the other day. Arrow indicates the start of the first administration of mepolizumab or placebo at day 4 after the first label intake. (D and E) and (G and H) A simple linear regression was performed for the uplabeling (day 0–7) and downlabeling phases (day 8–22) separately to compare elevation and intercepts with the axes. NS = not significant, ∗∗p ≤ 0.01.

After labeling with 6,6-^2^H_2_-glucose, 10 patients in the placebo group and 10 patients in the mepolizumab group were sampled at multiple days. Individual patients within each group were sampled on different days in order to have as much as possible information about eosinophil kinetics in time. The chosen time points were largely similar between the two treatment groups. Moreover, all patients underwent two separate labeling procedures: a first one at baseline (day 0; first phase) and a second one 84 days (12 weeks) after the first labeling procedure (second phase). During the course of the study, mepolizumab or placebo was administered every 4 weeks (28 days) with the first administration at day 4 after the first label intake (Figure 1). During the first uplabeling phase (from day 0 to day 7 after labeling intake, Figures 4C and 4D), the increase of ^2^H-label (“uplabeling”) originating from eosinophil DNA is similar for both placebo and mepolizumab treatments despite the fact that the number of blood eosinophils significantly decreased within 2–3 days of treatment. From day 8 onward, the loss of ^2^H-label (“downlabeling”) was significantly slower in eosinophils of the mepolizumab group than in the placebo group (linear regression, p = 0.0013, Figures 4C and 4E). Beyond day 32, the label in eosinophils dropped below the level of detection in both groups. Interestingly, the difference in downlabeling measured between placebo and mepolizumab groups during the first phase was not evident during the second phase (Figure 4F and H, p = 0.18). Thus, after 12 weeks of treatment with mepolizumab or placebo, the remaining eosinophils in the peripheral blood showed similar kinetics to those prior to treatment.

### The effect of mepolizumab on eosinophil kinetics in sputum

**Figure 5:**
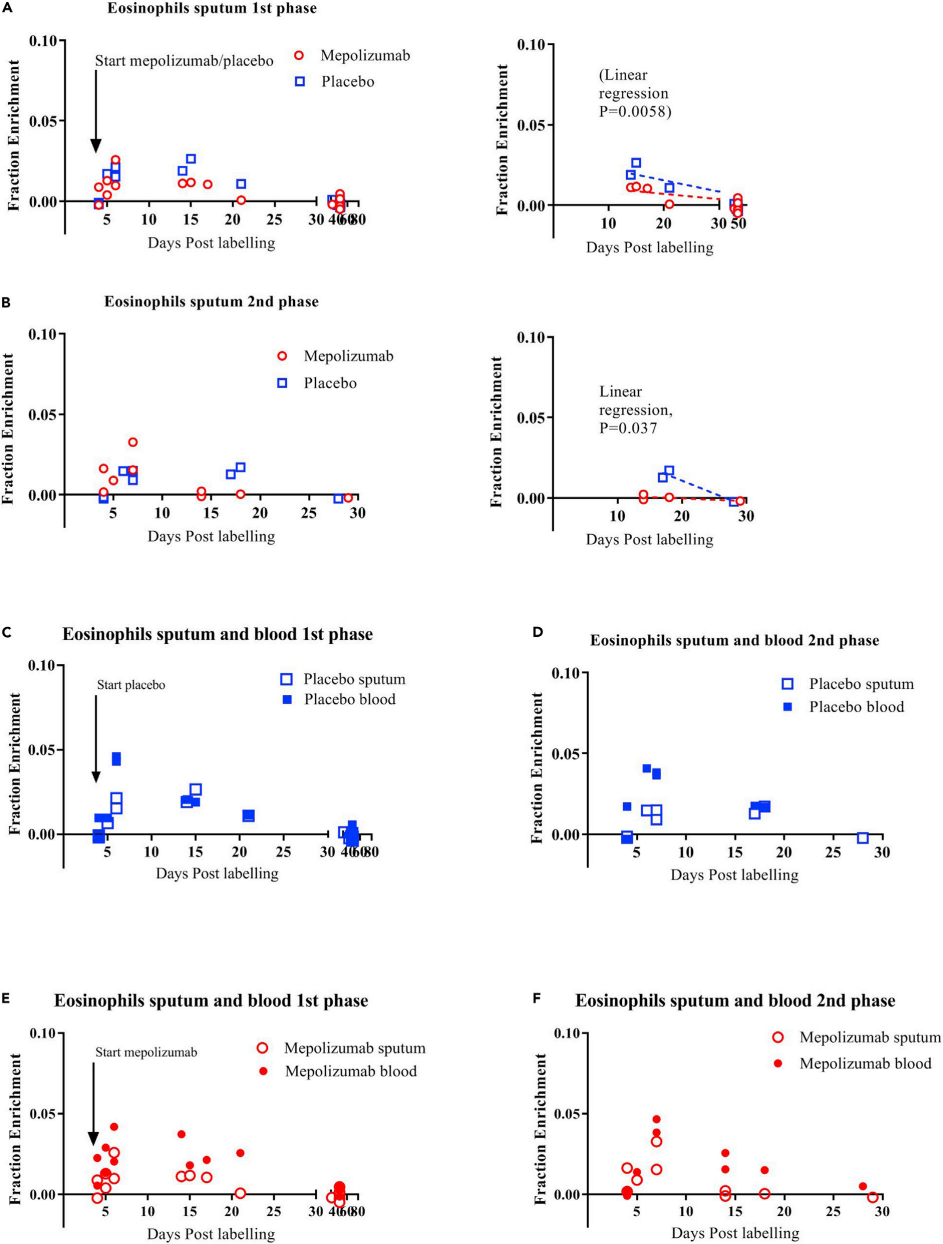
Deuterium enrichment of sputum eosinophil DNA and paired comparison to blood eosinophil DNA (A) ^2^H enrichment of DNA of sputum eosinophils was determined before label intake and at several days after label intake for patients in the placebo (blue) and the mepolizumab group (red) during the first labeling period. (B–F) (B) This was similarly done for sputum eosinophils during the second labeling period. The paired ^2^H enrichment of blood eosinophil (closed symbols) and sputum eosinophil (open symbols) DNA is shown for the placebo group during the first labeling (C) and second labeling period (D). This was also done for the mepolizumab group (closed is blood, open is sputum) during the first labeling period (E) and the second labeling period (F). Individual data points are shown. Arrow indicates the start of the first administration of mepolizumab or placebo at day 4 after the first label intake.

**Figure 6:**
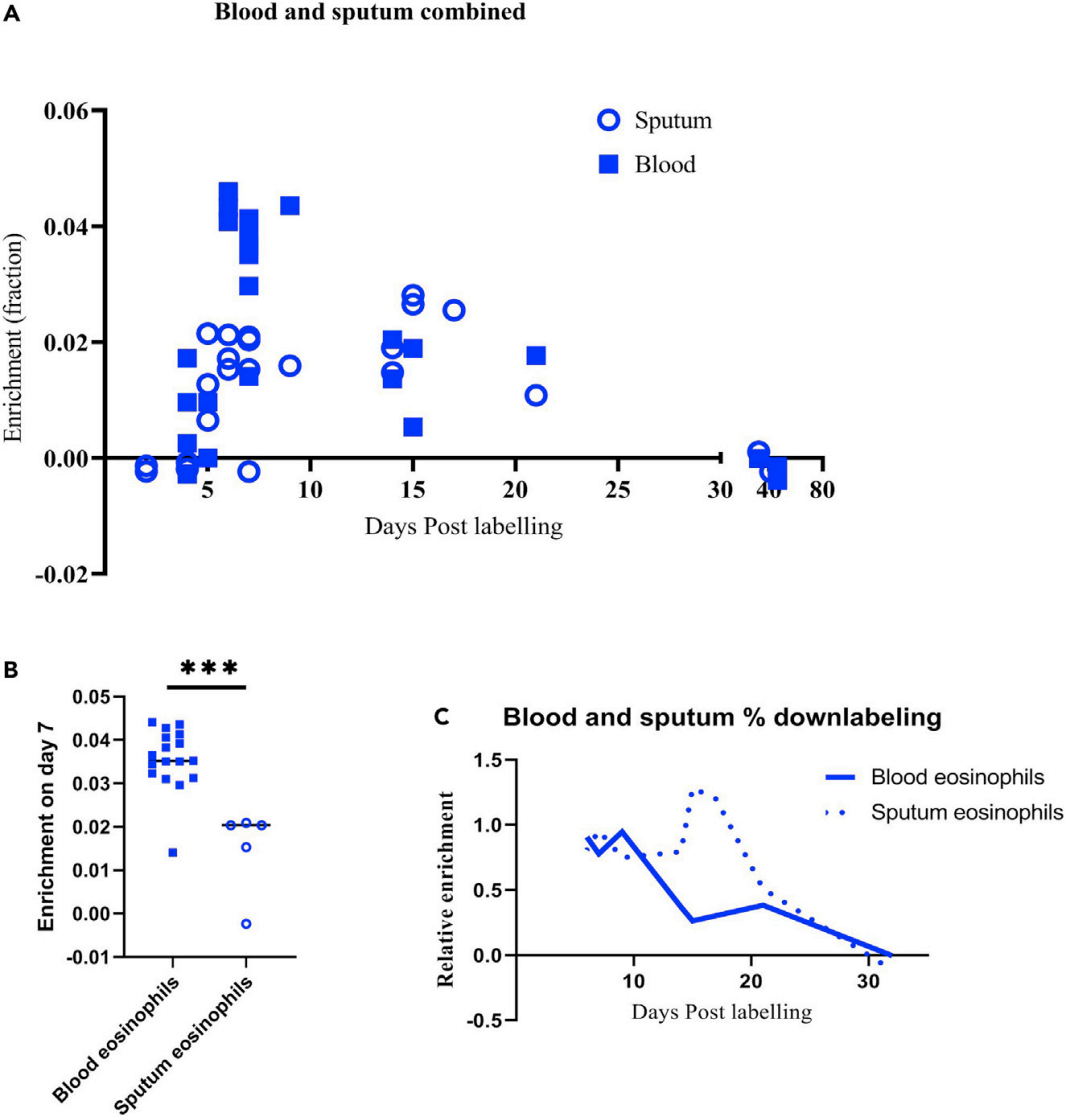
DNA ^2^H enrichment of paired sputum and blood granulocytes combined with a previous study (A) The data points of Figures 5C and 5D were combined and plotted together with data points of a similar study (EHLA study, n = 9). Open circles represent sputum samples, and closed squares represent blood samples. (B) DNA ^2^H enrichment on day 7 was plotted for both blood and sputum samples. (**C)** The ratio of DNA ^2^H enrichment on each day after day 7 (start of downlabeling) to the maximum DNA ^2^H enrichment on day 7 was calculated and plotted for blood (continuous line) and sputum (dotted line).

Sputum samples were taken either close to or far from the day of label intake because it was hypothesized that ^2^H enrichment in eosinophils would be particularly different during those periods in time after treatment with anti-IL-5 (see also Discussion). Indeed when sputum eosinophil enrichment was compared between the placebo and mepolizumab groups during the first phase, the mepolizumab group showed lower ^2^H-enrichment on day 14 and beyond (Figure 5A, linear regression, p = 0.0058). Similar to blood, deuterium label in the DNA of eosinophils from sputum on days close to the day of label intake (day 4–6) was not different between the two groups (p = 0.93). During the second phase of the study, again the upslope showed a very similar pattern (p = 0.93), but the loss of label seemed even more rapid in the mepolizumab group than in the placebo group (Figure 5B, p = 0.037). Next, we compared ^2^H-enrichment of sputum eosinophils with the blood eosinophils taken on the same day and from the same donor. This revealed that in the placebo group (Figures 5C and 5D) and mepolizumab group (Figures 5E and 5F), DNA from sputum eosinophils was overall lower enriched with deuterium compared to DNA from blood eosinophils. Because we had a low amount of successful ^2^H-enrichment data from sputum eosinophils, we included sputum and blood data from an earlier EA study (eosinophil half-life in the lungs of asthma patients (EHLA)-study, ABR NL44779.041.13). We combined these data with the data from both phases of the placebo group of this study to have as much as possible information about sputum eosinophil kinetics in patients with EA in the absence of mepolizumab (Figure 6A). The combined data set showed that sputum eosinophils might have reached a ^2^H-plateau between day 6 and day 17 after label intake before declining thereafter. In contrast, a distinct peak was observed in blood eosinophils at day 6–7 after label intake (Figure 6A). Furthermore, the height of enrichment was overall lower in the sputum than in blood (Figures 6B and 6C).

### Basophil kinetics in blood

**Figure 7:**
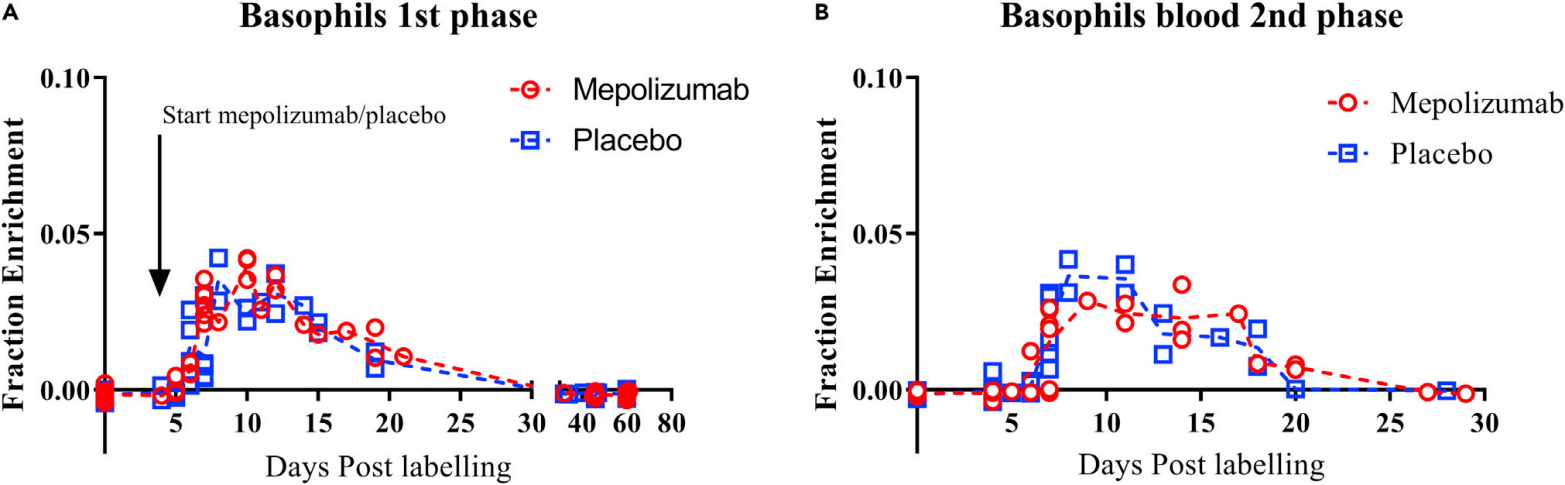
Deuterium enrichment blood basophil DNA (A) ^2^H enrichment of DNA of blood basophils was determined before label intake and at several days after label intake for patients in the placebo (blue) and the mepolizumab group (red) during the first labeling period. (B) DNA of blood basophils during the second labeling period. The individual data points are shown for each time point, and a dotted line connects the median values of each day. Arrow indicates the start of the first administration of mepolizumab or placebo at day 4 after first label intake.

The appearance of the first ^2^H-label in basophils in the blood was 6 days after label intake, which is considerably longer than that of the eosinophils that appeared 4 days after labeling (Figures 7A and 7B). A “plateau” of maximum enrichment was present from day 7–12 after label intake, contrasting a clear peak of enrichment found for blood eosinophils isolated from the same patients. After day 12, label disappeared at a similar rate as was seen in blood eosinophils, and from day 32 and beyond, no ^2^H-label was detected (Figure 7A). Interestingly, despite expression of a functional IL-5Rα, the kinetics of basophils in the mepolizumab group was similar to the kinetics of basophils seen in the placebo group during both phases of the study.

## Discussion

To our knowledge, this is the first study in which a nonradioactive, nontoxic, *in vivo* labeling method was used to study eosinophil and basophil kinetics in patients with EA. Moreover, this is also the first study in which these kinetics were directly compared between mepolizumab- and placebo-treated patients. The disadvantage of previous *ex vivo* labeling methods was the concomitant granulocyte activation or damage that in turn changed granulocyte homing characteristics (Farahi et al., 2012). The life cycle of granulocytes seems best described by a linear conveyor belt model in which a “first in, first out” principle is present such as suggested by Cartwright et al. in the 1960s (Cartwright et al., 1964). Unfortunately, for determination of the true life span of granulocytes using such a conveyor belt model, it is essential to obtain kinetic data of granulocyte progenitors in the bone marrow. No such data are available for eosinophils precluding a reliable estimation of the absolute life span of human eosinophils. Therefore, this study focused on relative differences in enrichment/kinetics between mepolizumab- and placebo-treated patients rather than the determination of changes in absolute life spans.

### The effect of mepolizumab on eosinophil kinetics in blood

The effects of mepolizumab on eosinophil kinetics were highly surprising. Despite a rapid decrease in the absolute numbers of eosinophils in the blood, the upslope of ^2^H-enrichment in the DNA of eosinophils in the mepolizumab group was unaltered compared with the placebo group. Even more unexpected was the significantly slower loss of ^2^H-enrichment in the DNA of eosinophils in the mepolizumab group. These data might be explained by at least four not mutually exclusive and even interacting hypotheses.

Lack of IL-5 leads to accumulation of eosinophils in the bone marrow. This hypothesis is supported by Palframan et al. (Palframan et al., 1998) showing the important role of IL-5 in the mobilization of eosinophils from the bone marrow. The retention of eosinophils induced by mepolizumab can explain the rapid (<2 days) decrease in eosinophil numbers which is faster than the postmitotic pool transit time of these cells (at least 4 days) (Figure 4A).

Lack of IL-5 leads to less dividing/differentiating progenitors. Despite an unaltered number of EoPs in the bone marrow (Menzies-Gow et al., 2003), a lower number of eosinophil progenitors proliferate in the bone marrow as a result of IL-5 blockade (Sanderson et al., 1985). This hypothesis fits with the finding that treatment with mepolizumab leads to lowering of mature eosinophils in the peripheral blood without a maturational arrest, i.e., the postmitotic transfer time is not affected (Figures 4C and 4D). This situation is also found in mice (Schollaert et al., 2014). Furthermore, this line of reasoning is also supported by our finding that the kinetics of uplabeling and the maximum enrichment of eosinophils is similar irrespective of the presence of anti-IL-5. The latter indicates that a less number of progenitors divide during treatment with mepolizumab, but they do so at the same rate as without mepolizumab treatment. A third possibility is the presence of multiple subsets of eosinophils (Marichal et al., 2017)^,^(Mesnil et al., 2016) which might be differentially sensitive to *short-term* treatment with mepolizumab. This difference in sensitivity might be at the level of the two earlier hypotheses. Based on our own data, it should be noted that such multiple subsets have identical uplabeling kinetics. In mice, a population of resident eosinophils has been reported. This population is different from blood eosinophils and is characterized by a dim IL-3Rα and a high CD62L expression (Mesnil et al., 2016). The fact that IL-3Rα expression decreased (Figure 3F), while CD62L expression increased (Figure S4A), after treatment with mepolizumab fits this hypothesis. On the other hand, an increase in CD62L is not completely surprising, as IL-5 has been shown to cause CD62L downregulation or shedding (Johansson, 2014). The same situation has been found for IL-3Rα expression as it was attenuated on eosinophils after treatment with mepolizumab (Kelly et al., 2017), although the underlying mechanism has not been elucidated.

A last possibility concerns the return of eosinophils from the tissues. It is unclear what percentage of eosinophils return to the peripheral blood after homing to the tissues and which part of the cells is cleared by macrophages in the tissue by efferocytosis (Felton et al., 2014). It is also unknown whether IL-5 plays a role in this balance. However, our data support enhanced return of these cells after an acute lowering of IL-5. It is possible that such returning eosinophils cause the delayed downlabeling of blood eosinophils. Because tissue eosinophils has been reported to be less sensitive to the effects of IL-5 (Liu et al., 2002) (Conus et al., 2010), it is possible that mepolizumab pushes the balance from IL-5-sensitive inflammatory blood eosinophils toward non-IL-5-responsive (tissue) eosinophils. Also, the finding that long-term treatment is not associated with a delay in blood enrichment fits this hypothesis as a very low number of eosinophils are left in the inflamed tissue (see the low eosinophil enrichment in the sputum after long-term treatment with mepolizumab Figure 5F).

Taken together, because mature eosinophils continue to be present in the blood and tissue with highly similar kinetics before and after treatment with mepolizumab, IL-5 is more likely a growth (proliferation) factor for mitotic IL-5Rα eosinophil progenitors than an essential factor for differentiation. The situation after prolonged treatment with mepolizumab is, therefore, similar as found in IL-5-knock-out (KO) mice (Foster et al., 1996). The differentiation of eosinophils *per se* is not affected, only the absolute number of dividing IL-5Rα^+^/CD34^-^ eosinophil progenitors (promyelocytes and myelocytes). This also explains the absence of reactive eosinophilia in both IL-5-KO-mice and long-term treatment with mepolizumab (Foster et al., 1996).

### Sputum eosinophils are longer lived than blood eosinophils

The number of successful (analyzable) samples of sputum eosinophils in our study is relatively low because of the low absolute numbers of eosinophils in the sputum, particularly in the mepolizumab-treated patients. Nonetheless, comparing blood and sputum ^2^H enrichment of the same donor in the placebo/EHLA groups (Table 1), the sputum data show a similar timing in uplabeling, but an overall lower maximum enrichment (Figures 6B and 6C). In other words, on the day of maximal enrichment, eosinophils isolated from sputum samples showed a statistically significant lower abundance of ^2^H-label (p < 0.001, Figures 6B and 6C). This finding points at the presence of long-lived nonlabeled eosinophils in the sputum (without mepolizumab) at the moment that labeled cells enter this compartment. This results in dilution of label because unlabeled DNA from eosinophils remaining longer in the airways will “dilute” the ^2^H-labeled DNA of eosinophils first appearing in the airways. This indicates that eosinophils remain longer in the airways than in blood. A sputum residence time is difficult to estimate, but it is less than 40 days because no labeled eosinophils are found in the sputum after this time.

### The effect of mepolizumab on eosinophil kinetics in the sputum

After treatment with mepolizumab, the situation in the airways is complex. Label disappearance in the sputum seems to be more rapid, especially after the second labeling period (after 80 days of treatment with mepolizumab). Thus, the low number of eosinophils found in sputum after treatment with mepolizumab cannot only be explained by slower homing of ^2^H-labeled eosinophils because this would result in a delayed upslope and longer presence of ^2^H label in the sputum, which is apparently not the case. An interesting hypothesis (discussed earlier) is the possibility that the absence of IL-5 pushes the balance between blood and inflammatory tissue eosinophils toward the blood.

Our study has consequences for the interpretation of old studies suggesting longevity of eosinophils in the lung tissue of patients with asthma (Flood-Page et al., 2003). The interpretation that eosinophils are long lived (>20 weeks) in the lung tissue of patients with asthma is not supported by our data showing complete disappearance of label in the sputum after 24–48 days. The alternative explanation of persistent tissue eosinophilia in patients with asthma treated with mepolizumab might be migration of EoPs toward the lungs followed by local maturation (Menzies-Gow et al., 2003). Another possibility is that eosinophils in the sputum are not representative of cells residing in the inflammatory lung tissue.

### The effect of mepolizumab on basophil kinetics

The number of basophils did not decline significantly upon treatment with mepolizumab (Figure 2B). This is in line with a recently published study (Wright et al., 2019). We have now also shown that basophil kinetics were not altered by treatment with mepolizumab. This is not completely surprising because even though basophils are responsive to IL-5 (Denburg et al., 1991) (Bischoff et al., 1990), their survival and proliferation have not been found to be mediated by IL-5, in contrast to IL-3 (Tanimoto et al., 1992). In a different study in which a higher dose of 250–750 mg of mepolizumab was administered, basophil numbers were slightly attenuated after at least 8 weeks of treatment in asthmatics (Buttner et al., 2003) (Nair et al., 2009), pointing at some redundancy between the βc-containing cytokine receptors for IL-5 and IL-3 (Martinez-Moczygemba and Huston, 2003). The increase in ^2^H-label enrichment in the DNA of basophils with a plateau on day 7–12 was much slower than in blood eosinophils. This indicates that basophils have progenitors that are more heterogeneous or slower with respect to their division rate and/or division time. The post mitotic pool transit time (PMPtt) of the fastest basophils (5–6 days) is slightly longer than that of eosinophils (4–5 days) which is not in line with previous published work in which the PMPtt was found to be equal to that of eosinophils (around 4.3 days) (Steinbach et al., 1979). This discrepancy might be explained by the fact that the study of Steinbach et al. enrolled only patients with cancer (Steinbach et al., 1979). It is possible that PMPtt of basophils is shortened by active systemic inflammation as has been found for neutrophils (Terashima et al., 1996).

### Conclusions

Treatment with mepolizumab does not delay the differentiation of eosinophils, but rather suppresses the number of proliferating IL-5Rα-expressing eosinophil progenitors. The remarkably slow downlabeling of eosinophils in blood is consistent with a modulating role of IL-5 in the balance of the presence of these cells between blood and tissue. The striking similarities between treatment with mepolizumab and the situation in IL-5-KO mice support the hypothesis that mepolizumab is particularly antagonizing reactive eosinophilia rather than blocking the maturation of the complete eosinophil compartment.

### Limitations of the study

Our study contains some potential caveats associated with the applied technology and the treatment with mepolizumab. The enrichment of DNA occurs during cell division in the bone marrow in eosinophil progenitors. After the last cell division, eosinophil precursors mature for at least 4 days in the bone before exiting to the peripheral blood. This technology allows the identification of differences in kinetics in the order of days rather than hours. Therefore, subtle differences in cellular kinetics evoked by an intervention in the order of hours can easily be missed. Another caveat is the analysis of kinetics of eosinophils in the sputum after long-term treatment with mepolizumab. The cell numbers were so low that only a limited number of accurate enrichment determinations could be performed.

## STAR★Methods

### Key resources table

**Table:** 

REAGENT or RESOURCE	SOURCE	IDENTIFIER
**Antibodies**		

Mouse anti-human CD16 PE-Cy7 (clone 3g8)	Biolegend	AB_314215 (Cat. No. 302015)
Mouse anti-human CD34 Alexa Fluor 700 (clone 581)	Biolegend	AB_2561494 (Cat. No. 343525)
Mouse anti-human CD206 PerCP/Cy5.5 (15-2)	Biolegend	AB_10900990 (Cat. No. 321121)
Mouse anti-human IL-3Rα Brilliant Violet 605 (clone 6H6)	Biolegend	AB_2562115 (Cat. No. 306025)
Mouse anti-human CD193 Alexa Fluor 647 (clone 5E8)	Biolegend	AB_2275699 (Cat. No. 310709)
Mouse anti-human CD62L PerCP/Cy5.5 (clone DREG-56)	Biolegend	AB_893396 (Cat. No. 304823)
Mouse anti-human CD14 APC-H7 (clone MPHIP9)	BD biosciences	AB_1645464
Mouse anti-human CD45 Krome Orange (clone J33)	Beckman Coulter	AB_a96416
Mouse anti-human IL-5Rα PE (clone 26815)	Thermo Fisher	AB_2608179
Mouse anti-human CD9 Cy2 (clone S32)	In house	N/A

**Chemicals, peptides, and recombinant proteins**		

LIVE/DEAD Fixable Violet Dead Cell Stain Kit	Thermo Fisher	Cat No. L34964
6,6-^2^H_2_-glucose	Cambridge Isotope laboratories	DLM-349-PK
Sodium acetate	Sigma – Aldrich	Cat. No. 71183
Acetic acid	Sigma – Aldrich	Cat. No. A6283
Zinc sulfate	Sigma - Aldrich	Cat No. Z4750
1 N NaOH, molecular biology grade	Sigma - Aldrich	Cat No. 72068
Acid phosphatase (potato, 1 kU)	Calbiochem	Cat No.80602-592
S1 nuclease	Sigma - Aldrich	Cat No. N5661
Pentafluorobenzyl hydroxylamine (1 mg ml−1 aqueous solution	Sigma - Aldrich	Cat No. 194484
Acetic anhydride	Sigma - Aldrich	Cat No. 242845-100G
N-methylimidazole	Sigma - Aldrich	Cat No. 336092
Sodium sulfate, granular, anhydrous	Sigma – Aldrich	Cat No. 23931-3
Dichloromethane	Sigma – Aldrich	Cat No. 66740
Ethyl acetate	VWR International	Cat No. 23882.296
Hydroxylamine HCl	Sigma – Aldrich	Cat No. 157417-100G
Pyridine	Sigma - Aldrich	Cat No. P57506
Human plasma albumin (albuman-40)	Sanquin plasma products	UMC Utrecht
Sputolysin	Merck Millipore	Cat. No. 560000-10ML

**Critical commercial assays**		

NucleoSpin Blood kit	Machery - Nagel	REF 740899.50

**Software and algorithms**		

GraphPad Prism 8.0	La Jolla	https://www.graphpad.com/scientific-software/prism/
FlowJo v10	LLC	https://www.flowjo.com/solutions/flowjo/downloads

### Resource availability

#### Lead contact

Further information and requests for resources and reagents should be directed to and will be fulfilled by the lead contact, prof. dr. Leo Koenderman (l.koenderman@umcutrecht.nl).

#### Materials availability

Materials used or generated in this study will be available upon reasonable request.

#### Data and code availability

Anonymized data are not freely accessible but can be obtained on reasonable request as they concern clinical human data. Any additional information required to reanalyze the data reported in this paper is available from the lead contact upon request.

### Experimental model and subject details

#### Subjects

A total of 20 patients with moderate to severe EA were recruited at the respiratory medicine departments of the University Medical Center in Utrecht (UMCU) and of the Haga Hospital in the Hague. All participants gave a written informed consent in accordance with the Declaration of Helsinki. The study was approved by the local institutional review board (IRB). All participants were aged between 18 and 70 years and were diagnosed with EA according to GINA (global initiative for asthma) guidelines by an experienced respiratory physician. For baseline characteristics of both groups, refer to Table 1. In short, patients had clinical features (episodic shortness of breath, often accompanied by cough and/or wheezing) and reversibility of the forced expiratory volume in 1s (FEV1) after treatment with 400 μg of salbutamol (≥12% or ≥200 mL compared with baseline). The eosinophilic phenotype of asthma was defined as at least one blood test within one year before enrollment with a minimal blood eosinophil concentration of 0.25 x 10^6^/ml. Patients with autoimmune diseases (e.g. rheumatoid arthritis), insulin-dependent diabetes, cancer, and chronic infection were excluded. Smokers and patients with proven allergic bronchopulmonary aspergillosis (ABPA) were also excluded.

Because of a low number of successful isolations of sputum eosinophils, we added data from an earlier study in which 9 patients with EA were enrolled (EHLA study, NL44779.041.13). These patients had similar inclusion and exclusion criteria to this study (Table 1), but were not randomized for treatment with mepolizumab or placebo. They were kept on their usual treatment regimen.

#### Randomization of subjects and drug administration

After enrollment and the first labeling procedure, patients were blindly randomized by the pharmacy of the UMCU, using an automated randomization application. The individual patient was numbered sequentially and assigned to either group before the patient entered the study. The randomization was performed for placebo (n = 10) or 100 mg of mepolizumab (n = 10). Injections were administered every 4 weeks (28 days) subcutaneously in the upper arm for a total of 4 times (Figure 1). The first administration of mepolizumab or placebo was 4 days after deuterium labeling. In order to estimate the sample size, we used a simple T-test with an alpha of 0.05, a beta of 0.20 (power of 0.8), and an effect size of 0.3 (this was estimated based on previous nonpublished data). This power analysis generated a sample size of n = 8 for both groups.

### Method details

#### Deuterium labeling procedure and sampling

All participants were *in vivo* labeled with deuterium-labeled glucose (^2^H). (Macallan et al., 1998). Before each labeling procedure, a baseline blood sample was taken via venepuncture. This sample was used to determine the baseline DNA enrichment. Thereafter, 12 equal doses of metabolic-grade 6,6-^2^H_2_-glucose amounting to a total of 1 g/kg bodyweight (Cambridge Isotope Laboratories, Tewksbury, MA, USA) were administered orally every half hour. Each time 1/12^th^ of the total weight was aliquotted in a disposable cup. We minimized normal glucose intake during the labeling procedure (maximum of 200 kcal each time with 15 g of carbohydrate and 8 g of fat). *In vivo*, 6,6-^2^H_2_-glucose is metabolized and incorporated into the DNA of only dividing cells via the de novo nucleotide synthesis pathway (Macallan et al., 2009). During the labeling procedure, three small blood samples were obtained via finger pricks on filter papers to determine the availability of label in plasma (plasma glucose enrichment) (Macallan et al., 2009). This was done at 35 minutes, at 185 minutes, and at 305 minutes after the 1^st^ dose. Gloves were worn, and the individual’s hands were cleaned each time to prevent contamination from the cups on the filter paper. The labeling procedure was performed twice per patient: at the start of the study (t= 0 days, phase 1) and after 80 days (t=0 days, phase 2), see Figure 1. For all participants, a total of 10 blood samples were obtained, 2 prior to both labeling procedures and 8 other time points (Figure 1). In addition, three sputum samples per patient were obtained. For blood and sputum, the time points varied between volunteers, but were largely similar between the mepolizumab and placebo group to maximize the total number of data points for each group.

#### Blood collection and processing

At each time point (see Figure 1), 18 ml of blood was collected in sodium heparin tubes (Vacuette® Greiner bio-one, Kremsmünster, Austria), via venepuncture. Absolute granulocyte count was measured using a fully automatic hematology analyzer, CELL-DYN Emerald (Abbott, Chicago, IL, USA). Subsequently, erythrocytes were lysed using an ice-cold lysis buffer (150 mM NH_4_Cl, 10 mM KHCO_3_, and 0.1 mM NA_2_EDTA): 18 ml of blood was mixed with 32 ml of this buffer for approximately 15 minutes at 4°C. After this, the remaining leukocytes were washed and resuspended in a staining buffer (PBS2+) consisting of PBS supplemented with 0.32% w/v trisodium citrate (prepared by the pharmacy of the UMCU) and 10% w/v human plasma albumin solution (Sanquin, Amsterdam, the Netherlands). After washing the cells twice with 10 ml of PBS2+, they were resuspended in 400 μl of PBS2+ and kept on ice until further staining.

#### Sputum induction and processing

Sputum was induced with inhalation of 3-5% saline aerosols, for 5 minutes each time until successful expectoration. The induction was performed using a clinical nebulizer (DeVilbiss Ultraneb 2000 ultrasonic nebulizer) with a maximal output of 2.5 ml/min (Rutgers et al., 2000). The aerosols were administered to the subjects through a 100-cm long tube with an internal diameter of 22 mm and inhaled via the mouth. Subsequently, the patients inhaled hypertonic saline aerosols for 3 periods of 5 min each. After each inhalation, or as soon as the subjects experience cough, they were asked to blow their nose, to rinse their mouth and throat with water, and to expectorate sputum into a clean plastic container by coughing. Thereafter, the collected sputum was weighed and kept on ice until further processing. The samples were afterward incubated with 4:1 (w/vol) 0.1% sputolysin (dithiothreitol) solution in PBS with a corrected osmolarity. The osmolarity was corrected, by adding saturated saline solution until an osmolarity was reached of 280-290 mOsm (Merck Millipore, Darmstadt, Germany). Thereafter, sputum was homogenized with a Pasteur pipette and placed in a 37ºC water bath for 20 minutes (Rutgers et al., 2000). The resulting cell suspensions were then filtered through a 40-μm filter (Corning, New York, USA) and washed with cold (4°C) PBS. For all sputum samples, cytospin slides were obtained and stained with May-Grünwald-Giemsa (standard protocol). The remaining sample was kept on ice after which it was later used for granulocyte isolation as described in the following.

#### Granulocyte isolation from sputum and blood

In PBS, suspended sputum samples were stained with a live/dead violet cell staining kit in a concentration of 1:1600 at room temperature for 10 minutes (Table 2). After washing, cells were suspended in 400 μl of PBS2+ and stained with the following antibodies (Table 2): CD16-PE/Cy7 (1:100), CD34-Alexa Fluor 700 (1:100), CD206-PerCP/Cy5.5 (1:100), IL-3Rα-Brilliant Violet 605 (1:100), CD14 APC-H7 (1:100), CD45-Krome Orange (1:50), IL-5Rα-PE (1:100), and CD9-Cy2 (1:100). Lysed blood was similarly stained in 400 μl of PBS2+ but without the live dead stain, CD45 and CD206. Instead, CD193-Alexa Fluor 647 (1:50) and CD62L-PerCP/Cy5.5 (1:100) were used to stain blood samples. Usually 250.000 cells were isolated using an AriaII or ARIAIII FACS sorter (BD Biosciences, Mountain view, CA, USA). Blood granulocytes were identified based on doublet exclusion, FSC/SSC properties, and CD14^-^ (Figure S1 for gating strategy). Subsequently, eosinophils were identified as SSC^high^, CD16^-^, CD193^+^, and CD9^+^. Sputum cells were gated similarly, with addition of a Live/DEAD stain and CD45 stain to exclude necrotic cells and nonleukocytes (Figure S2). EoPs were identified as low FSC, CD14^-^, CD34^+^, and IL-5Rα^+^ (Mori et al., 2009). Sorted populations were reanalyzed (typically >99% pure). Cellular DNA was isolated from fresh samples using a NucleoSpin Blood kit according to producers' instructions (Machery-Nägel, Oensingen, Switzerland). DNA was stored at -20°C until further processing.

#### Determination of DNA ^2^H enrichment

After collection of all samples, DNA was enzymatically hydrolyzed into deoxyribonucleotides (Busch et al., 2007). DNA samples were first suspended in water with a volume of 200 μl each. The samples were thereafter mixed with 50 μl of a hydrolysis cocktail which contains a mix of sodium acetate (concentration of 375 nM), zinc sulfate (concentration of 1.33 nM), acid phosphatase enzyme (concentration of 5U/mg), and S1 nuclease enzyme (concentration of 0.5 U/μl). See Table 2 for more information regarding the different chemicals. The obtained deoxyribonucleotides were derivatized to pentafluoro triacetate (PFTA) derivatives.(Busch et al., 2007). To do so, samples were first transferred to a glass vial after which 100 μl of a fresh prepared solution of 1 mg/ml pentafluorobenzyl hydroxylamine (Table 2) in water was added and vortexed. Thereafter, 75 μl of glacial acetic acid was added and the samples were again vortexed and placed in a 100°C oven for 30 minutes. After cooling, 1 ml of acetic anhydride was added, along with 100 μl of cold (4°C) N-methylimidazole and samples were directly vortexed. The reaction was allowed to proceed for 15-20 minutes after which 2 ml of water was added. Samples were again vortexed. After that, 750 μl of dichloromethane (DCM) was added to the vials and the phases were allowed to separate for 1 minute. The organic layer was then transferred to a new and clean vial. These vials were dried overnight, using a Speedvac, at room temperature. The dried samples were resuspended in 100 μl of ethyl acetate and transferred to a gas chromatography (GC) column. These columns were then also dried in the Speedvac for approximately 1 hour after which the dried samples were resuspended in 50 μl of ethyl acetate and capped immediately thereafter. The quantity of ^2^H-labeled and unlabeled adenosine derivatives was measured on an Agilent 7980A/5975C GC-MS in negative chemical ionization mode scanning for m/z 435 (M+0, unlabeled) and m/z 437 (M+2, labeled). The resulting enrichments were corrected for natural background enrichment using enrichment of the baseline blood samples. They were also corrected for the availability of 6,6-^2^H_2_-glucose in plasma. Plasma deuterium enrichment was performed by transferring at least two blood drops from the filter paper into a 1.5-ml centrifuge tube, and 50% (vol/vol) ethanol was added thereafter. This was left at ambient temperature for 30 minutes after which it was vortexed and transferred to another Eppendorf tube. These tubes were centrifuged rapidly at t >15,000 g for 10 min at ambient temperature. The supernatant was transferred to another tube once more, and the samples were dried inside an Eppendorf concentrator at 45°C. The dried samples were resuspended in a fresh 25-μl solution of 1% (wt/vol) hydroxylamine/HCl in pyridine (1 mg per 100 μl). These samples were heated inside a dri-block for 60 minutes at 100°C. Thereafter, samples were once more dried inside the Eppendorf concentrator set at 45°C for at least 2 hours. After the samples were cooled, 25 μl of acetic anhydride was added, and the samples were left at ambient temperature for 20 minutes. After heating the samples for 10 minutes at 70°C, the derivatization step was completed. The supernatant was dried once more inside the Eppendorf concentrator at 45°C after which the samples were resuspended in 100 μl of ethyl acetate and transferred to GC vials. The vials were analyzed inside GC-MS using selective ion monitoring for ions of m/z 328 and 330 (Macallan et al., 2009). Finally, deuterium enrichment was also corrected for de novo synthesized/obtained by base salvage ratio which was shown to be 0.65 (Macallan et al., 2009).

### Quantification and statistical analysis

The enrichments of PFTA was measured, using the peak areas for ions m/z 435(m) and m/z (m+2) of the PFTA. Thereafter, the tracer to trace ratios (TTR) = (m+2)/m were calculated. The ratio (m+2)/ (m) of abundance-matched samples were used to calculate the enrichment of deuterated deoxyadenosine, calibrating against standard curves of known enrichment. A similar method was used to calculate deuterium enrichment in plasma glucose. FlowJo v10 (LLC, Ashland, OR, USA) was used to analyze the flow cytometry data (see Figures S1 and 2 for the gating strategies). Median fluorescence intensity was calculated for several proteins on the surface membrane on the gated eosinophils or basophils. Data were expressed as median with interquartile range, unless otherwise stated. All statistical analyses were performed using GraphPad Prism 8.0. In these analyses, a p-value of p < 0.05 was considered statistically significant. Each figure legend appoints to which statistical test was performed and the value of *n*.

### Additional resources

Additional information about the FOOTSTEP trial (clinical registry number: EudraCT: 2016-002014-52.) can be found at: https://www.clinicaltrialsregister.eu/ctr-search/trial/2016-002014-52/NL. Local IRB number: ABR NL57535.041.16. The graphical abstract was created with BioRender.com.

## Supporting citation

The following references appear in the Supplemental information: Sehmi et al., 2016.
