# Supplementary material for: Differential effects of short- and long-term treatment with mepolizumab on eosinophil kinetics in blood and sputum in eosinophilic asthma
Source: iScience. 2021 Jul 28;24(8):102913. doi: 10.1016/j.isci.2021.102913 (PMC8361259; doi:10.1016/j.isci.2021.102913)
Supplement: Document S1. Figures S1–S4 [file mmc1.pdf]

iScience, Volume *24*

Supplemental information

Differential effects of short- and long-term treatment with mepolizumab on eosinophil kinetics in blood and sputum in eosinophilic asthma

Marwan Hassani, Tamar Tak, Corneli van Aalst, Saar van Nederveen, Kiki Tesselaar, Nienke Vrisekoop, and Leo Koenderman

Figure S1 related to figures 2-7.

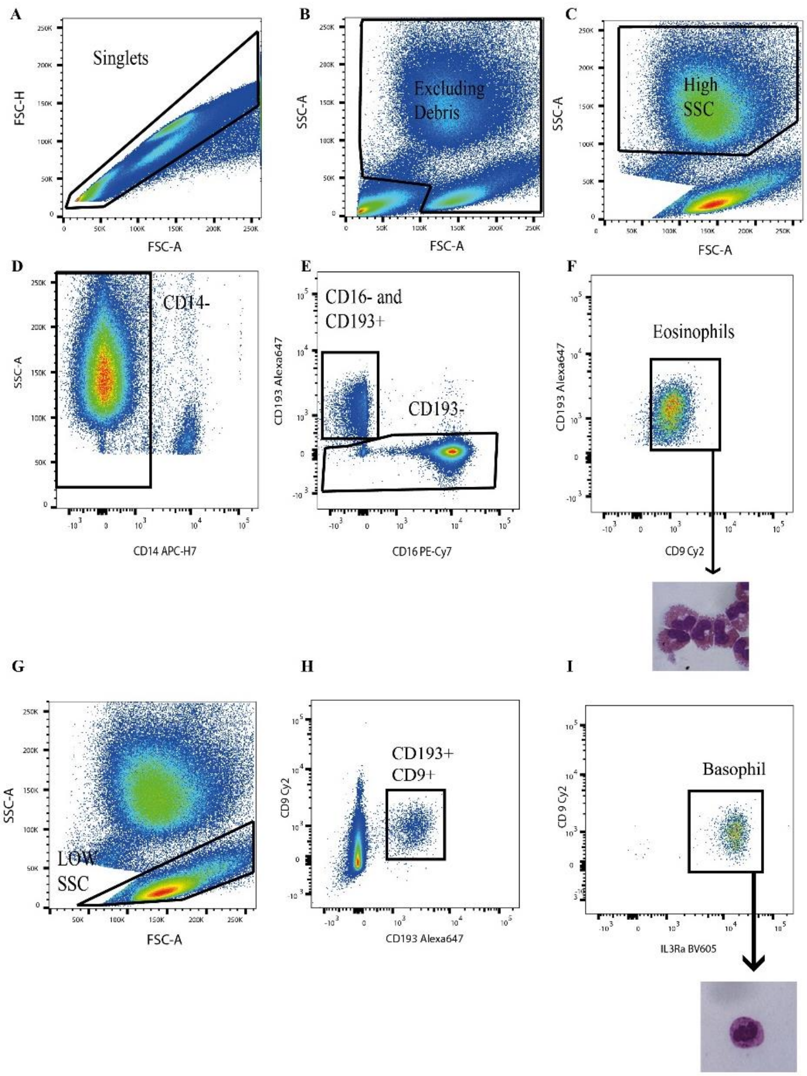

Figure S1. Gating strategy for FACS sorting eosinophils and basophils in the blood.

*Blood eosinophils were identified by first gating on singlets on the basis of FSC-A and FSC-H (**A**), debris is excluded on the basis of FSC-A and SSC-A (**B**), subsequently only high SSC-A cells are gated (**C**), CD14^{high} cells are excluded (**D**), CD16^{neg} and CD193^{pos} cells are gated (**E**) and finally within the later gate CD9^{pos} cells are gated (**F**). These cells were FACS sorted and cytospins were stained with May-Grünwald Giesma (MMG) and microscopically evaluated. An example of a representative microscopic image (objective 100x) of a cytospin of sorted eosinophils is shown. Basophils were sorted from the same samples, but this time SSC^{low} cells are gated (**G**), followed by CD9^{pos} and CD193^{pos} (**H**) and finally gated for IL-3Rα^{high} to obtain basophils (**H**). Also a representative (MMG stained)microscopic image (objective 100x) of sorted basophils is shown.*

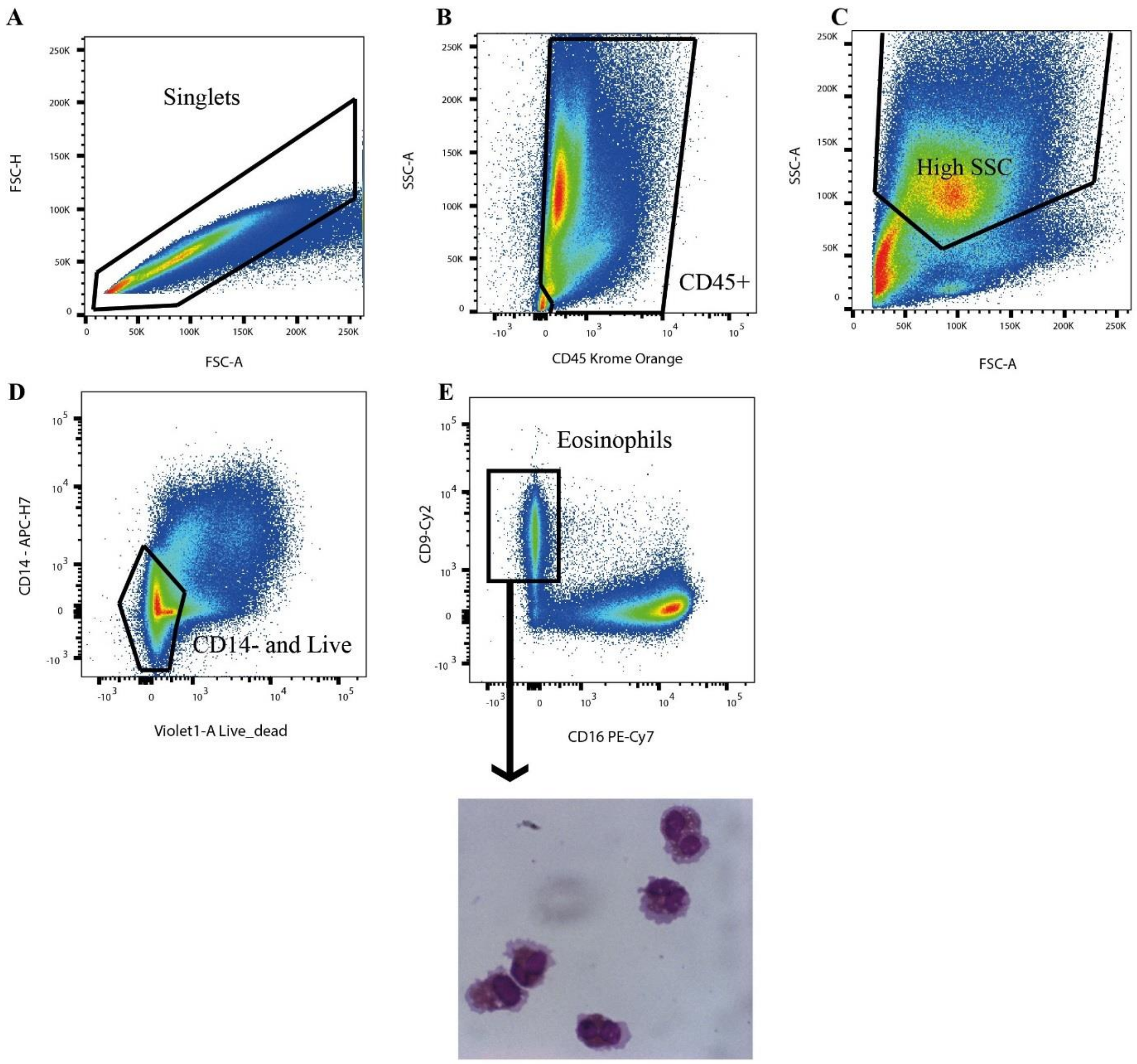

Figure S2 related to figures 2-7.

Figure S2. Gating strategy for FACS sorting eosinophils in the sputum.

*Sputum eosinophil were gated after only gating singlets (**A**), CD45^{pos} cells (**B**), SSC^{high} cells (**C**) and CD14^{neg} and negative for live/dead stain (**D**). Finally eosinophils were gated on the basis of CD16^{neg} and CD9^{pos} cells and FACS sorted **(E)**. A representative microscopic image (100x objective) of a MMG stained cytospin of sorted eosinophils is shown.*

Figure S3 related to figures 2 and 3.

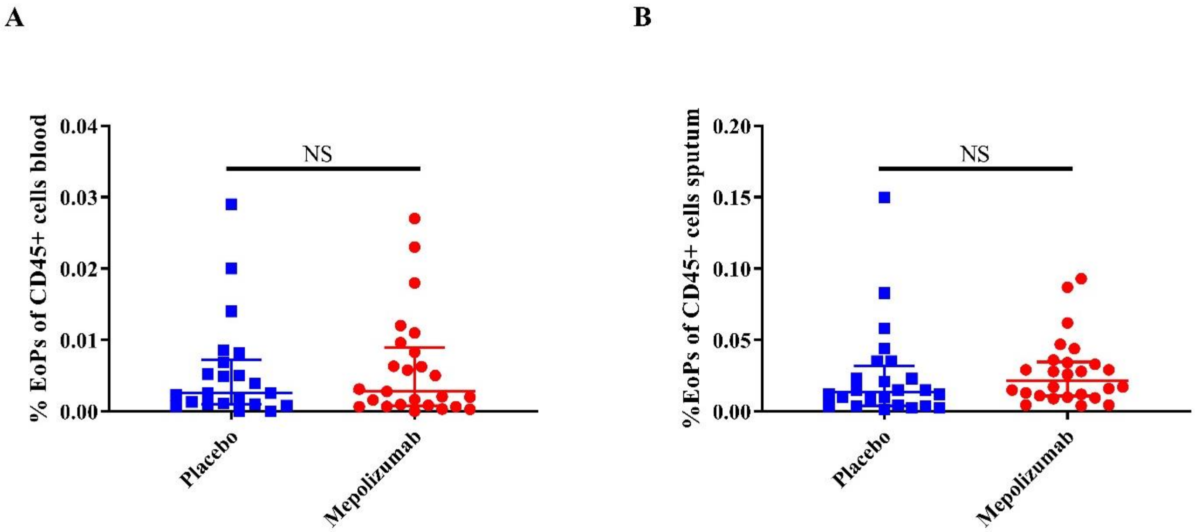

Figure S3. Number of EoPs in blood and sputum.

*EoPs in the blood (**A**) and sputum (**B**) were gated and enumerated as a percentage of total white blood cells as was published before(Mori et al., 2009),(Sehmi et al., 2016). Single data points with median and interquartile range are shown for mepolizumab n= 23 (red) and placebo n=25 (blue). A Mann-Whitney test was performed to compare both groups. NS not significant.*

Figure S4 related to figures 2 and 3.

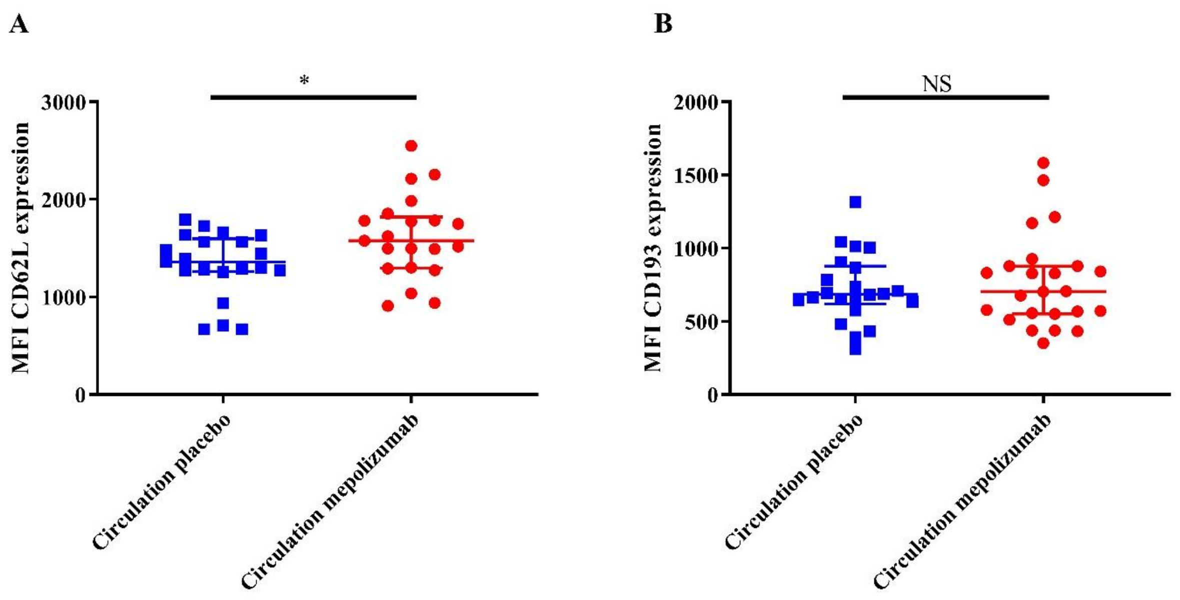

Figure S4. Median fluorescence intensity for CD62L and CD193 in blood eosinophils.
*The MFI for CD62L membrane expression (**A**) and CD193 membrane expression (**B**) is plotted for multiple samples after at least 3 days of treatment with placebo, n= 25 (blue) or mepolizumab, n=23 (red). A Mann-Whitney test was used to compare both treatment groups. Individual data points with median and interquartile range is shown. NS not significant and * P≤ 0.05.*
